# Assessment of the Toxic Effects of Heavy Metals on Waterbirds and Their Prey Species in Freshwater Habitats

**DOI:** 10.3390/toxics10110641

**Published:** 2022-10-25

**Authors:** Jeganathan Pandiyan, Arumugam Poiyamozhi, Shahid Mahboob, Khalid A. Al-Ghanim, Fahad Al-Misned, Zubair Ahmed, Irfan Manzoor, Marimuthu Govindarajan

**Affiliations:** 1 Department of Zoology and Wildlife Biology, A.V.C. College, Mannampandal 609 305, Mayiladuthurai, Tamil Nadu, India; 2 Department of Zoology, College of Science, King Saud University, Riyadh 11451, Saudi Arabia; 3 Department of Biology, Indiana University, Bloomington, IN 47405-7000, USA; 4 Unit of Mycology and Parasitology, Department of Zoology, Annamalai University, Annamalainagar 608 002, Tamil Nadu, India; 5 Unit of Natural Products and Nanotechnology, Department of Zoology, Government College for Women (Autonomous), Kumbakonam 612 001, Tamil Nadu, India

**Keywords:** aquatic habitats, freshwater lake, pollution, heavy metals, waterbirds, prey species, human health and management

## Abstract

Waterbirds may be a good indicator of harmful metal levels in aquatic environments. Waterbirds’ organs and tissues were tested for the presence of pollutants, such as metals. However, very few reports describe the use of bird feathers and their prey in metal analysis. In the present research, seven metals were measured in the tissue, kidney, liver, and feathers of the Indian pond heron, the black-crowned night heron, and their prey species, including crabs, prawns, molluscs, and fishes from a freshwater lake. Metals were examined using an ECIL-4141-double beam atomic absorption spectrophotometer (DB-AAS). Metal concentrations differed considerably in the tissue, kidney, liver, and feathers of the Indian pond heron and black-crowned night heron (*p* < 0.001). Indeed, this research discovered a good correlation between the metals of prey species and the tissues, kidneys, liver, and feathers of waterbirds that were tested. The regression model explained that the *Cyprinus carpio* influence the accumulation of metals about 98.2% in tissues, *Macrobrachium rosenbergii* and *Cyprinus carpio* around 86.3% in the kidney, the *Labeo rohita* almost 47.2% in the liver and *Labeo rohita* nearly 93.2% on the feathers of the Indian pond heron. On the other hand, the *Mystus vittatus*, *Cyprinus carpio*, *Labeo rohita* influence about 98.8% in tissue, the *Claris batrachus* and *Tilapia mossambica* around 93.3% in kidney, the *Mystus vittatus*, *Cyprinus carpio*, about 93.2% in liver and the freshwater crab (*Travancoriana schirnerae*), freshwater prawn (*Macrobrachium rosenbergii*) and a fish (*Cyprinus carpio*) nearly 93.2% in feathers in the black-crowned night heron. This research evaluated metals in the dead carcasses of waterbirds, a non-invasive biomonitoring technique for pollution. Overall, the investigation revealed that the lake is severely contaminated with metals. Therefore, the management and protection of aquatic habitats, particularly freshwater lakes, should be enhanced to rescue wild species that rely on aquatic ecosystems and to ensure that people have access to clean drinking water.

## 1. Introduction

Globally, aquatic environments are declining owing to diverse environmental contamination, notably metal pollution [1,2,3,4]. Heavy metals are harmful toxic substances that contribute to environmental pollution because they alter the physiology and behavior of diverse living forms, particularly top predators in an ecosystem [5,6]. The adverse effects of heavy metals on waterbirds include increased reproductive failures and susceptibility to bird illnesses [7,8]. Waterbirds are considered a model organism for detecting water contamination [9]. Consequently, waterbirds are considered bio-indicators [10,11], mostly for heavy metals [12]. Heronries are principally concerned with analyzing various heavy metals since herons are the top predators in the food chain and have wide home ranges in aquatic ecosystems [9,13].

In light of the importance of the liver and kidney in metal detoxification, the present research evaluated metal levels in waterbirds’ feathers, liver, and kidneys. Due to their toxicity, lead (Pb), nickel (Ni), mercury (Hg), chromium (Cr), arsenic (As), copper (Cu), and zinc (Zn) have all been the subject of substantial research [14]. Because of the potential for metals to have a wide range of negative consequences for waterbirds, assessing metal levels in birds’ muscles has received a fair amount of interest [15,16]. Indeed, there is a lack of studies in India that evaluate metals in waterbirds using tissues, organs, or feathers, such as the sixteen species studied in one research that used organs [17] and the two studies that used liver and kidneys in Nilgiris, India [15,16]. Although bird feathers have been used to evaluate metal exposure [10,18,19], very few studies have been conducted in India using bird feathers [11,19,20,21,22].

Despite this, waterbirds’ feeding ecology and dietary preferences led to the accumulation and bio-magnification of heavy metals [23]. The evaluation of metals in waterbird prey species is also not well-covered in India, except for a small number of researches focusing on shorebirds [19,20,21,22]. Metals in waterbirds need to be assessed, since environmental contamination has put several waterbird species at risk [24]. Consequently, the current study focuses on determining the level of heavy metals in the tissues, liver, kidney, and feathers of heronry waterbirds and their prey species, such as crustaceans, molluscs, and fishes in the Veeranam Lake, which is one of the largest lakes in Southeast Asia and provides drinking water for human society as well as seasonal feeding and breeding grounds for numerous species of waterbirds.

## 2. Materials and Methods

### 2.1. Study Area

**Figure 1:**
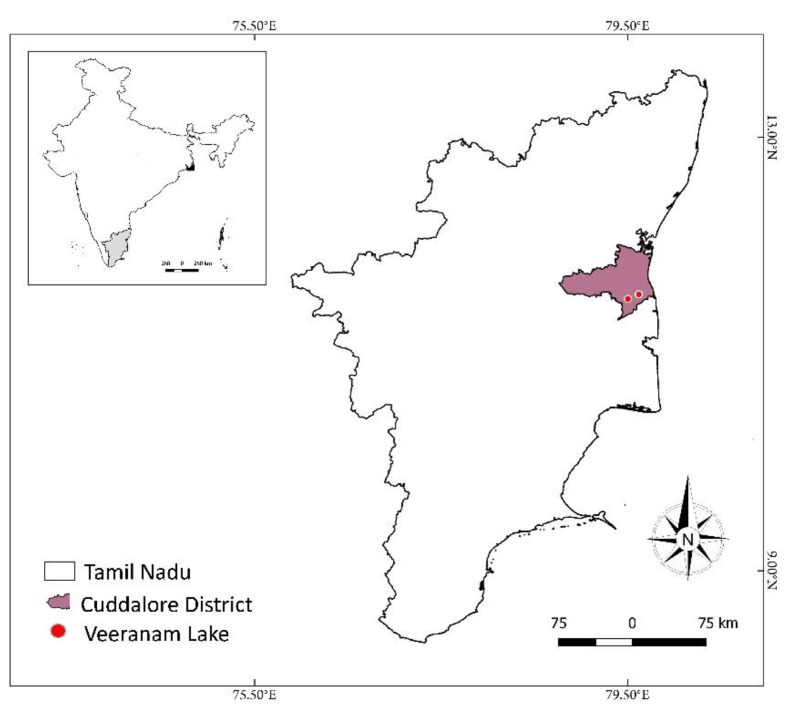
Map showing the location of Veeranam freshwater Lake, Tamil Nadu, India.

The location of the Veeranam Lake is 11°21′07.2″ N 79°32′03.1″ E, Tamil Nadu, India (Figure 1). The lake is 30 km from the Bay of Bengal. During the ninth century, the lake was dug by the Chola King, “King Paranthaga”. It is possible to store 930 Mcf of water inside the lake’s 25 km^2^ area. The lake’s 34 sluices yearly provide water to 40,000 acres of agricultural farmland. Vadavar and Sengal Stream are the two largest rivers that take water from the Keealani (Lower Anaicut) of the Cauvery River and discharge into the Veeranam Lake. Every day, 7000 ft^3^ of water is discharged for agricultural use in the lake’s neighboring communities. The major crops grown by farmers near the lake are *Oryza sativa*, *Saccharum officinarum*, *Arachis hypogaea*, *Gossypium species*, *Abelmoschus esculentus*, *Solanum tuberosum*, *Capsicum* sp., *Musa* sp., *Solanum* sp., etc. The lake offers a range of microhabitats that serve as feeding and nesting grounds for several waterbird species [25]. The lake has a subtropical climate. The lake had temperatures between 30 °C and 38 °C [26]. The Veeranam lake is an important water reservoir, and it supplies drinking water to the capital of Tamil Nadu, India; hence, the lake is both socially and ecologically significant. Moreover, the lake is home to various seasonal, resident migratory and migratory waterbirds and their prey species; as a result, it is regarded as a significant worldwide aquatic environment [27].

### 2.2. Assessment of Metals

Seven different metals, such as arsenic (As), chromium (Cr), copper (Cu), lead (Pb), mercury (Hg), nickel (Ni), and zinc (Zn), were assessed from the various organs, including tissues, liver, kidney, and feathers of dead carcasses of the Indian pond heron (*Ardeola grayii*) and black-crowned night heron (*Nycticorax nycticorax*), and their prey species such as freshwater crab (*Travancorianas chirnerae*), freshwater prawn (*Macrobrachium rosenbergii*), and fishes such as *Claris batrachus*, *Mystus vittatus*, *Cyprinus carpio*, *Labeo rohita* and *Tilapia mossambica* [19].

### 2.3. Collection of Crabs, Prawn and Fish Samples

Various types of fish and prawns were collected from the lake at six random locations using a 20 × 15 m fishing and cast net with a mesh size of 2.3–2.8 cm. The fish and shrimp species in the lake were collected from three hectares of each random location. Each randomized location yielded nine distinct samples. Nine individuals from each species of fish and prawn were used for the metal analyses. The fish and prawns were weighed using their wet weight, and the samples were transported to the laboratory in an icebox at 30 °C. Crabs were captured using a fishing drag net (30 × 10 m), and crab samples were preserved in an icebox for subsequent examination [19].

### 2.4. Digestion of Crabs, Prawn and Fish Samples

Metals were analyzed using the internal soft muscles of fish, prawn, and crab species. Six of each kind of fish, prawn, and crab were prepared for testing. In a polystyrene tube, 25 g of tissue samples were collected from prey animals. Each polystyrene tube contained nine samples that were dried at 50 °C to a consistent weight and cooled at room temperature (25 °C), along with samples that were prepared to the nearest 0.1 mg. Thus, 1 mL of nitric acid (HNO_3_) was added to each sample, which was then kept at 25 °C for one hour. Again, it was heated to 50 °C for four hours to facilitate prey animal digestion. In addition, 100 mL of H_2_O_2_ was added to each tube, which was then heated at 50 °C for an additional hour to complete the prey sample digestion process [21]. The final digested samples were transferred to a clean vial for additional metal detection using ECLC-4141-double beam atomic absorption spectrophotometer (AAS) [21].

### 2.5. Collection and Digestion of Tissues, Liver, Kidney, and Feathers of Waterbirds

Three grams of tissues, liver, and kidney were dissected from the dead carcasses of the *Ardeola grayii*, and *Nycticorax nycticorax* during our field work in the lake and kept in a sterilized tube at −20 °C until digestion. The samples’ digestion was performed using the microwave digestion unit, using 69% HNO_3_ 10 mL for ten min, 70% HClO_4_ one mL for five min, and 30% H_2_O_2_ 5 mL for 10 min at 250 W magnetron power settings. Standard filter paper was used to filter the digested samples, which were kept in sterilized polythene vials in the freezer [11]. The primary feathers were collected from dead carcasses of waterbirds from the lake for the metals analysis [4]. Double distilled water was used to wash the external metals and other chemicals. Washed feathers were allowed to dry for about twenty-four hours at 60 °C in the oven and it was collected after reaching constant dry mass and secured at 0.001 g [21]. Consequently, the nitrogenous acid with 65% and perchloric acid with 70% mixture was used at the ratio of 4:1 ratio to digest the feathers [28]. Later, double distilled water was added up to 10 mL and the samples were kept in a metal-free vial at 20 °C to assess metals [21].

### 2.6. Analytical Procedure and Quality Control (QC)

Quality control (QC) is a process used to determine standards and respective guidelines; QC samples were used for every 15 samples to ensure the instrument’s stability for the accurate assessment of the various metals in the waterbirds’ tissues, liver, kidney, feathers, and prey organisms. The differences in the concentrations of metals in the quality samples were less than fifteen percent. In each set of studies, blank reagents were utilized to guarantee the amount of contamination. For subsequent statistical analysis, a mean of nine duplicates was used for each evaluation. The analytical precision procedure is disclosed as relative standard deviation (RSD) with a range of 5% to 10%, by estimating SD/mean for better accuracy of the analytical procedures. The calibration curves were prepared individually for each metal at standard solution concentration through various PPM, 0.5, 1, 2, 5 and 10. Nevertheless, the standard daily procedure also provides (SSS) dilution by combining HNO_3_ with 65% and H_2_O_2_ as well as H_2_O with 30% (*v*/*v*/*v* = 1:1:3). Consequently, the calibration curve and its relative coefficient for limit of detection (LOD) were generated. In order to ensure accurate readings of the samples, the instrument was calibrated with a blank to establish zero [10,21].

A minimum of nine samples were examined for each metal, and the mean results were utilized for further statistical analysis. All chemicals, including reagents and stock solutions, were purchased from Merck, in order to provide a wide spectrum of analytical detectability. The glassware was washed with deionized water and nitric acid (30%). The metal evaluation was conducted using a double-beam atomic adsorption spectrophotometer (AAS). Using Perkin-Elmer Pure Plus multi-element standards, standards were developed [11,19,21].

### 2.7. Data Analysis

The data were validated, analyzed and calculated using descriptive statistics, including arithmetic mean and standard error for every variable; values are represented as mean ± standard error. The data entry was carried out using MS Excel [29] and the entire analysis was carried out using SPSS 25.0 [25]. ANOVA was employed to test the various hypotheses in the study, particularly the differences of metals studied from the various organs of the waterbirds and their prey matters. Correlational analysis was carried out among the metals of different organs of waterbirds with their prey species. Multiple regression was applied to predict the metals’ sources in waterbirds, which the prey species used as predictor variables. To test the various hypotheses *p* < 0.05, *p* < 0.01 and *p* < 0.001 were used and interpreted with standard procedures [30].

## 3. Results

### 3.1. Metals in Waterbirds

Pb levels were higher in tissues, kidneys, and feathers, whereas Cr levels were higher in the Indian pond heron and black-crowned night heron, according to the research (Table 1 and Table 2). The Indian pond heron had Pb > Cr > Ni > Zn > As > Cu > Hg, whereas the black-crowned night heron contained Pb > Cr > As > Zn > Cu > Ni > Hg. The metal concentrations in the examined tissues and organs of the Indian pond heron and black-crowned night heron differed substantially (*p* < 0.001).

### 3.2. Metals in Prey Species

In the prey animals, Pb was higher (8.48 ± 0.234 ppm) in the crab (*Travancoria naschirnerae*) and the prawn species (*Macrobrachium rosenbergii*) (5.56 ± 0.171 ppm), and the As was greater in *Claris batrachus* (13.04 ± 0.038 ppm). However, the *Mystus vittatus* and *Cyprinus carpio* showed the highest Cr (5.75 ± 0.142 ppm). The Pb was higher in *Labeo rohita* and *Tilapia mossambica* (5.74 ± 0.073 ppm) and (5.76 ± 0.056 ppm), respectively. The metals varied significantly among the prey species studied (*p* < 0.001). The level of metals in the prey species were Hg > As > Cr > Zn > Ni > Cu > Pb (Table 3).

### 3.3. Relationship of Metals between the Prey Species and Waterbirds

The level of metals in tissues, kidneys, liver and feathers of the Indian pond heron and black-crowned night heron positively correlated with the metals of their prey species (*p* < 0.001) (Table 4 and Table 5).

The results of the regression explained that, in the Indian pond heron, *C. carpio* influenced the accumulation of metals by about 98.2% (F = 1262.102, *p* < 0.000) in the tissues; the *Macrobrachium rosenbergii* and *C. carpio* showed 86.3% (F = 73.542, *p* < 0.000) in the kidney; the *L. rohita* influenced metal accumulation in the liver of 47.2% *(F =* 19.943, *p* < 0.000); and the *L. rohita* influenced about 93.2% (F = 2218.307, *p* < 0.000) in the feathers (Table 6).

The *M. vittatus*, *C. carpio*, *L. rohita* influenced the accumulation of metals in the tissues of the black-crowned night heron by about 98.8% (F = 146,846, *p* < 0.000). The *C. batrachus* and *T. mossambica* explained about 93.3% of the accumulation of metals in the kidney. The *M. vittatus*, *C. carpio* influenced the level of metals in the liver by about 93.2% (F = 79.864, *p* < 0.000) and the freshwater crab (*T. naschirnerae*), freshwater prawn (*M. rosenbergii*) and the fish (*C. carpio*) explained 93.2% (F = 106.158, *p* < 0.000) of the accumulation of metals in the feathers of black-crowned night heron (Table 7). Overall, the accumulation of metals in the waterbirds influenced by their prey is significant.

## 4. Discussion

The inland wetland ecosystems not only provide acceptable habitats for aquatic organisms, but also provide enough drinking water for people, making their presence very significant. For instance, fifty percent of wetland habitats are vanishing and the remaining wetland habitats are eroding, presumably owing to intense human pressures [31]. Nevertheless, the size and deterioration of wetland habitats considerably affect the number of migratory bird populations [22]. The majority of wetland ecosystems are polluted by a variety of contaminants, particularly metals; as a result of metal pollution, waterbirds are severely impacted by severe physiological deficiencies and behavioral issues [20,32]. In fact, the present research demonstrated a larger buildup of heavy metals in the tissues, organs, and feathers of Indian pond herons and black-crowned night herons than in earlier studies [18,33,34]. In addition, researchers have shown that the accumulation of metals in the bodies of waterbirds, such as Pb, Cr, Ni, Zn, As, Cu, and Hg, causes significant health concerns [8,13,35].

### 4.1. Lead (Pb)

In fact, lead (Pb) levels were greater in the tissues, liver, kidneys, and feathers of both species compared to other waterbird species tested in various regions of the world [9,24,33]. A study conducted in India revealed that the amount of Pb in various waterbirds was lower than in the present research [21,22]. Rather than other sources, dietary sources of metals in bird communities have been cited in studies [36]. In truth, the Indian pond heron and the black-crowned night heron eat mostly fish, although they may also consume crabs and molluscs when the fish density or quantity in their feeding grounds is low [37]. According to studies, fish, molluscs, and crustaceans have higher metal concentrations in their body [21,38]. Similarly, the present research indicated that the Pb concentration in fishes, molluscs, and crustaceans was much higher than in other species (Table 3). According to a study [24], the mating success of waterbirds is somewhat impacted by the excessive deposition of lead. Excess buildup of Pb in birds has also been linked to increased cellular stress resulting from metabolic inhibition of protein, lipids, and carbs [39]. The research highlighted the memory loss reported in birds as a result of elevated Pb levels in waterbirds [40].

### 4.2. Chromium (Cr)

Chromium (Cr) is sourced in the aquatic ecosystem through human activities such as domestic, small, medium and large-scale factories and agro-farm activities [41]. The study found that the Cr was lower than those studied in other parts of the world [42]; however, a greater concentration of Cr has been reported in various species of waterbirds [43]. A study reported that dietary preferences could enrich the higher exposure of Cr in the waterbirds [35]. A higher level of Cr was reported in fishes [21]; these waterbirds mainly feed on fish, which might be why the Cr was greater in the two species of waterbirds studied. In fact, the liver of the Indian pond heron and the black-crowned night heron showed a greater level of Cr 8.27 ± 0.02 and 6.98 ± 0.10, respectively (Table 1 and Table 2), which is an alarming concentration [10]. The threshold level of Cr is (2.8 lg/g), but the current study showed a greater level that might harm waterbirds [9]. A previous report stated that the greater level of Cr in birds could affect their DNA and protein, which will negatively affect the inheritance of characters [44].

### 4.3. Nickel (Ni)

The research discovered higher levels of Ni in the feathers and liver of both bird species examined. The highest concentration of Ni was obtained from a fish species (*C. batrachus*), a primary food source for the Indian pond heron and the black-crowned night heron [45]. Similarly, Ni was higher in the lake-dwelling crab studied. As secondary and tertiary consumers in a food chain/food web, fish and crustaceans exhibited a greater Ni concentration in aquatic ecosystems, according to studies [46]. Ni levels in two species of waterbirds and their prey are above the threshold limits [10]. The buildup of Ni in birds over the average concentration may impair their cellular communication, flying mechanics, and tumor development, resulting in increased mortality [13,47].

### 4.4. Zinc (Zn)

The feathers of the Indian pond heron and liver of the black-crowned night heron have a higher zinc concentration. In fact, the sources of zinc in birds may come from their food [42]. The research also discovered that prey matter had the highest levels of zinc, particularly crustaceans and fishes. According to a study, the feeding strategy of waterbirds influences zinc accumulation [21]. Species from different regions of the world [13,18] were found to have a higher concentration of zinc than those in the present research. However, feathers and different tissues of waterbirds were also shown to have lower levels of zinc [48]. However, a bigger Zn buildup might impact avian populations’ metabolic processes, resulting in significant bird mortality [49]. Moreover, Zn poisoning has been linked to various malformations in bird species [8].

### 4.5. Arsenic (As)

The effects of arsenic on birds have revealed a number of crucial difficulties [50]. According to the research, the liver of the Indian pond heron, the kidney of the black-crowned night heron, and their prey likewise had elevated levels of arsenic (Table 1, Table 2 and Table 3). The research discovered that the As concentration in the feathers, tissues, and other organs was greater than in other species tested [51]. In addition, their food species, particularly fish (*C. batrachus*), showed a greater amount of arsenic compared to prior fish studies [52]. As’s toxicity involves alterations in inter- and intra-cellular kinetic mechanisms, which result in ROS stress [51] and aberrant genomic expression in waterbirds [53]. The elevated levels of arsenic in waterbirds may also affect the mitochondria, which influence respiration and numerous physiological disorders [54].

### 4.6. Copper and Mercury (Cu-Hg)

In fact, the amount of Cu in the tissues, various organs, and feathers of both species of waterbirds is estimated to be lower in different locations of the world [13,50,55,56]. In this investigation, the prey species exhibited a normal Cu range. However, much study has been conducted to collect the Cu in the lake and the species that rely on it. In addition, the concentration of Hg in the feathers and other organs of the examined waterbirds was lower.

### 4.7. Relationship of Metals between the Prey and Organs of the Waterbirds

The metals of prey species exhibited good correlations with the tissues, liver, kidney, and feathers of the two examined species of waterbirds (*p* < 0.001) (Table 4 and Table 5). The prawn sp., *C. carpio*, and *L. rohita*, also caused metals to build up in the tissues, liver, kidney, and feathers of the Indian pond heron in about 98.2%, 86.3%, 47.2%, and 93.2% of the cases, respectively (Table 6). In general, the accumulation of metals in waterbirds might be influenced by their foraging strategies, food availability, and dietary preferences. However, the amount of metals in a bird may be regulated by metabolic processes, and the metabolic systems may govern the absorption and digestion of harmful compounds through their physiological activities. The amount of metals in birds is controlled by their detoxifying process, according to research [57]. However, the bioaccumulation and biomagnification of metals in biota are assisted by the interaction between prey and predators in a specific ecosystem, such that trophic systems may enhance metals in top predators [58].

Similarly, *M. vittatus*, *C. carpio*, and *L. rohita* explained approximately 98.8%, *C. batrachus* and *T. mossambica* explained 93.2%, *M. vittatus*, *C. carpio*, and *T. mossambica* explained about 93.2%, and crab, prawn species, and *C. carpio* defined approximately 93.2% of metals in the two examined species of waterbirds (Table 7). This research suggests that the buildup of metals in heronry waterbirds may be due to their dietary habits. According to studies, the eating habits of waterbirds may cause them to collect larger quantities of metals [59]. Consequently, the lentic environment encourages the larger accumulation of metals in aquatic creatures due to the limited daily movements and foraging distance, notably for fish, amphibians, crustaceans, and other prey species [60]. The waterbirds also fed on the available prey species in the lake, which may explain why both species had elevated metal levels in their bodies.

In fact, the amount of metals such as As, Cr, Cu, Pb, Hg, Ni, and Zn found in the tissue, liver, kidney, feathers, and prey of waterbirds in the present research exceeds the Indian and United States Environmental Protection Agency’s threshold values [61,62]. According to the current research findings, the quantity of metals extracted from the lake is alarming, and adequate rules must be implemented to protect aquatic environments.

## 5. Conclusions

In accordance with the current research findings, the levels of metals in the tissues, liver, kidney, feathers, and prey species of waterbirds are over the permissible limits indicated by the USEPA and ISI. The current investigation reveals an alarmingly high percentage of metals in the lake, which serves as a warning about its quality. In reality, the Veernanam lake supplies drinking water to the surrounding human population. The lake also provides drinking water to Chennai, the 7.5 million-person capital of the Indian state of Tamil Nadu. Additionally, the lake irrigates hundreds of hectares of agricultural land with water, yearly. In addition, the primary source of metals in the lake is the Thalai Cauvery, which the Cauvery River carries. Along the Cauvery River are many small, medium, and large-scale enterprises, including tanneries, battery factories, and distilleries. In addition, the river conveys wastewater from the river basin’s communities. Additionally, the river is laden with sewage from the local panchayat, cities, and municipalities. In addition, the Cauvery basin is renowned for its agricultural techniques, in which farmers apply enormous quantities of pesticides, fertilizers, and chemicals to seasonally cultivated crops.

Thus, the Veeranam Lake collects contaminants, together with river water, the main source of contamination. During the research periods, predators and waterbirds may have had higher metal concentrations. In fact, several studies [25,26,27] indicate that the lake of Veeranam serves as a feeding and nesting habitat for many waterbirds yearly. Nonetheless, the contaminated water harms both animal and human health, since the lake serves as a source of potable water for a substantial population. Globally, preserving a freshwater environment is essential for conserving aquatic-dependent biota [63,64]. In addition, a considerable study must be conducted on aquatic habitats to improve the quality and management of aquatic habitats, since they provide clean drinking water to human civilization and serve as a habitat for many species of flora and fauna.

## Figures and Tables

**Table 1:** Level of metals in the different organs of the Indian pond heron, Veeranam Lake, Cuddalore, District, Tamil Nadu. [Values are mean and SE; ppm (N = 3)].

Metals	Tissue	Kidney	Liver	Feather
As	0.10 ± 0.0001	0.20 ± 0.001	0.44 ± 0.007	0.09 ± 0.005
Cr	1.12 ± 0.03	1.07 ± 0.004	8.27 ± 0.02	0.71 ± 0.008
Cu	0.05 ± 0.003	0.06 ± 0.03	0.13 ± 0.001	0.28 ± 0.004
Pb	4.85 ± 0.09	5.50 ± 0.24	4.96 ± 0.03	4.60 ± 0.03
Hg	0.30 ± 0.002	0.10 ± 0.001	0.02 ± 0.0008	0.01 ± 0.0008
Ni	0.56 ± 0.02	0.71 ± 0.01	0.89 ± 0.01	0.96 ± 0.03
Zn	0.39 ± 0.01	0.19 ± 0.005	0.66 ± 0.01	1.37 ± 0.18

**Table 2:** Level of metals in the different organs of the black-crowned night heron, Veeranam Lake, Cuddalore, District, Tamil Nadu [Values are mean and SE; ppm (N = 3)].

Metals	Tissue	Kidney	Liver	Feather
As	1.92 ± 1.46	3.04 ± 0.31	2.63 ± 0.04	0.43 ± 0.007
Cr	0.72 ± 0.004	1.62 ± 0.13	6.98 ± 0.10	2.25 ± 0.09
Cu	0.54 ± 0.03	0.15 ± 0.08	0.51 ± 0.01	0.84 ± 0.63
Pb	5.39 ± 0.03	4.07 ± 0.69	5.63 ± 0.08	5.53 ± 0.05
Hg	0.01 ± 0.003	0.15 ± 0.13	0.04 ± 0.01	0.02 ± 0.007
Ni	0.54 ± 0.03	0.16 ± 0.08	0.57 ± 0.02	0.63 ± 0.08
Zn	1.26 ± 0.02	0.23 ± 0.08	1.41 ± 0.01	0.92 ± 0.01

**Table 3:** Metal accumulation in various prey species of waterbirds, Veeranam Lake, Tamil Nadu, India (Values are mean and SE; ppm).

Metals	Crabs (N = 6)	Prawn Species (N = 6)	*Claris batrachus* (N = 6)	*Mystus vittatus* (N = 6)	*Cyprinus carpio* (N = 6)	*Labeo rohita* (N = 6)	***Tilapia*** ***mossambica*** **(N = 6)**	*p* Value
As	5.58 ± 0.029	2.06 ± 0.06	13.04 ± 0.038	1.79 ± 0.036	2.45 ± 0.378	2.29 ± 0.298	0.43 ± 0.002	*p* < 0.001
Cr	1.81 ± 0.039	0.34 ± 0.010	9.70 ± 0.100	5.75 ± 0.142	3.02 ± 0.112	0.85 ± 0.079	0.35 ± 0.004	*p* < 0.001
Cu	3.60 ± 0.190	2.49 ± 0.186	1.83 ± 0.052	0.51 ± 0.015	0.11 ± 0.029	0.12 ± 0.008	0.008 ± 0.003	*p* < 0.001
Pb	8.48 ± 0.234	5.56 ± 0.171	4.86 ± 0.103	2.61 ± 0.107	6.88 ± 0.108	5.74 ± 0.073	5.76 ± 0.056	*p* < 0.001
Hg	0.05 ± 0.0006	0.13 ± 0.064	0.28 ± 0.072	0.10 ± 0.004	0.10 ± 0.047	0.05 ± 0.031	0.01 ± 0.004	*p* < 0.001
Ni	2.43 ± 0.039	0.50 ± 0.017	5.03 ± 0.027	0.79 ± 0.088	1.18 ± 0.383	0.23 ± 0.028	0.91 ± 0.024	*p* < 0.001
Zn	2.99 ± 0.006	1.34 ± 0.032	3.68 ± 0.092	2.82 ± 0.091	2.73 ± 0.120	1.70 ± 0.095	1.86 ± 0.059	*p* < 0.001

**Table 4:** Pearson Correlation of different metals assessed between the various organs of the Indian pond heron (*Ardeola grayii*) and their prey species (bold letter with * indicate the level of significance is *p* < 0.05; with ** indicates the level of significance at *p* < 0.001).

Variables	Tissue	Kidney	Liver	Feather	Crab	Prawn	*Claris batrachus*	*Mystus vittatus*	*Labeo rohita*	***Tilapia*** ***mossambica***	***Cyprinus carpio***
Tissue	1										
Kidney	0.939 ^**^	1									
Liver	0.593 ^**^	0.643 ^**^	1								
Feather	0.993 ^**^	0.936 ^**^	0.548 ^**^	1							
Crab	0.898 ^**^	0.884 ^**^	0.438 ^*^	0.903 ^**^	1						
Prawn	0.851 ^**^	0.898 ^**^	0.398	0.861 ^**^	0.956 ^**^	1					
*C. batrachus*	0.067	0.051	0.377	0.031	0.260	0.062	1				
*M. vittatus*	0.903 ^**^	0.752 ^**^	0.690 ^**^	0.886 ^**^	0.755 ^**^	0.631 ^**^	0.263	1			
*L. rohita*	0.984 ^**^	0.905 ^**^	0.603 ^**^	0.979 ^**^	0.908 ^**^	0.829 ^**^	0.187	0.935 ^**^	1		
*T. mossambica*	0.977 ^**^	0.917 ^**^	0.527 ^**^	0.978 ^**^	0.946 ^**^	0.887 ^**^	0.168	0.882 ^**^	0.984 ^**^	1	
*C. carpio*	0.991 ^**^	0.912 ^**^	0.511 ^*^	0.995 ^**^	0.905 ^**^	0.850 ^**^	0.047	0.896 ^**^	0.985 ^**^	0.983 ^**^	1

**Table 5:** Pearson correlation of different metals assessed between the various organs of the night heron (*Nycticorax nycticorax)* and their prey species (bold letter with ** indicates the level of significance at *p* < 0.001).

Variables	Tissue	Kidney	Liver	Feather	Crab	Prawn	*Claris batrachus*	*Mystus vittatus*	*Labeo rohita*	***Tilapia*** ***mossambica***	***Cyprinus carpio***
Tissue	1										
Kidney	0.939 ^**^	1									
Liver	0.860 ^**^	0.883 ^**^	1								
Feather	0.925 ^**^	0.887 ^**^	0.906 ^**^	1							
Crab	0.927 ^**^	0.913 ^**^	0.792 ^**^	0.897 ^**^	1						
Prawn	0.868 ^**^	0.833 ^**^	0.702 ^**^	0.884 ^**^	0.956 ^**^	1					
*C. batrachus*	0.135	0.316	0.380	0.104	0.260	0.062	1				
*M. vittatus*	0.882 ^**^	0.867 ^**^	0.943 ^**^	0.844 ^**^	0.755 ^**^	0.631 ^**^	0.263	1			
*L. rohita*	0.968 ^**^	0.937 ^**^	0.919 ^**^	0.944 ^**^	0.908 ^**^	0.829 ^**^	0.187	0.935 ^**^	1		
*T. mossambica*	0.987 ^**^	0.955 ^**^	0.875 ^**^	0.939 ^**^	0.946 ^**^	0.887 ^**^	0.168	0.882 ^**^	0.984 ^**^	1	
*C. carpio*	0.978 ^**^	0.918 ^**^	0.855 ^**^	0.937 ^**^	0.905 ^**^	0.850 ^**^	0.047	0.896 ^**^	0.985 ^**^	0.983 ^**^	1

**Table 6:** A simple regression model explains the influence of various prey species on the accumulation of metals in tissues, kidneys, liver and feathers of Indian pond heron, Veeranam Lake, Tamilnadu, India.

Predictor Variables	Tissue-Pond Heron			Kidney-Pond Heron			Liver-Pond Heron			Feathers-Pond Heron		
	B	SE B	β	B	SE B	β	B	SE B	β	B	SE B	β
Crab	-	-	-	-	-	-	-	-	-	-	-	-
Prawn	-	-	-	0.455	0.192	0.397	-	-	-	-	-	-
*C. batrachus*	-	-	-	-	-	-	-	-	-	-	-	-
*M. vittatus*	-	-	-	-	-	-	-	-	-	-	-	-
*C. carpio*	0.776	0.022	0.991	0.355	0.105	0.565	-	-	-	-	-	-
*L. rohita*	-	-	-	-	-	-	0.458	0.103	0.690	0.674	0.014	0.995
*T. mossambica*	-	-	-	-	-	-	-	-	-	-	-	-
R	98.3%			87.5%			47.5%			94.1%		
R^2^	98.2%			86.3%			47.2%			93.2%		
ANOVA	(1,23), 1262.102, *p* < 0.000			(2,23) 73.542, *p* < 0.000			(3,23) 19.943, *p* < 0.000			(3,23) 2218.307, *p* < 0.000		

**Table 7:** The simple regression model explains the influence of various prey species on the accumulation of metals in tissues, kidneys, liver and feathers of black-crowned night heron, Veeranam Lake, Tamil Nadu, India.

Predictor Variables	Tissue-Night Heron			Kidney-Night Heron			Liver-Night Heron			Feathers-Night Heron		
	B	SE B	β	B	SE B	β	B	SE B	β	B	SE B	β
Crab	-	-	-	-	-	-	-	-	-	0.456	0.193	0.621
Prawn	-	-	-	-	-	-	-	-	-	0.922	0.248	0.729
*C. batrachus*	-	-	-	0.131	0.045	0.160	-	-	-	-	-	-
*M. vittatus*	0.269	0.128	0.284	-	-	-	0.417	0.173	0.470	-	-	-
*C. carpio*	−0.950	0.339	−1.176	-	-	-	1.176	0.432	1.557	0.474	0.291	0.903
*L. rohita*	1.016	0.362	0.952	-	-	-	-	-	-	-	-	-
*T. ossambica*	-	-	-	0.723	0.043	0.929	1.132	0.293	1.399	-	-	-
R	97.5%			93.8%			94.4%			94.1%		
R^2^	98.8%			93.2%			93.2%			93.2%		
ANOVA	(3,23), 146,846, *p* < 0.000			(2,23) 158.214, *p* < 0.000			(3,23) 79.864, *p* < 0.000			(3,23) 106.158, *p* < 0.000		

## Data Availability

Not applicable.
